# Time-restricted feeding enhances cross-tissue temporal coordination of mitochondrial-associated transcripts

**DOI:** 10.1016/j.isci.2026.115957

**Published:** 2026-05-15

**Authors:** Dylan C. Sarver, Yaniv Maddahi, Christopher S. Colwell, Aldons Jake Lusis

**Affiliations:** 1 Department of Medicine, Division of Cardiology, University of California, Los Angeles, Los Angeles, CA 90095, USA; 2 Department of Microbiology, Immunology, and Molecular Genetics, University of California, Los Angeles, Los Angeles, CA 90095, USA; 3 Department of Human Genetics, University of California, Los Angeles, Los Angeles, CA 90095, USA; 4 Department of Psychiatry and Biobehavioral Sciences, David Geffen School of Medicine, University of California, Los Angeles, Los Angeles, CA 90095, USA

**Keywords:** biological sciences, systems biology, transcriptomics

## Abstract

Coordinating metabolism with the day-night cycle is essential for health. Circadian rhythms synchronize cellular and tissue functions with the external environment. Nutrient timing is a potent circadian cue that entrains peripheral clocks across organs. Mitochondria exhibit daily rhythms in energy metabolism and redox regulation; however, the temporal organization of mitochondrial-associated transcriptional programs across tissues remains unknown. We hypothesized that time-restricted feeding (TRF) enhances the cross-tissue coordination of mitochondrial-associated transcripts (MATs). Using a 22-tissue, 24-h transcriptomic dataset from mice under *ad libitum* or TRF conditions, we applied correlation- and phase-based analyses to quantify the intra- and inter-tissue alignment of MAT expression. TRF markedly increased cross-tissue coordination, nearly quadrupling the number of globally aligned MATs. Among these, *Coq10b* emerged as the most rhythmically aligned gene across organs, highlighting it as a representative marker of coordinated MAT expression. Together, these findings reveal a previously unrecognized temporal organization of mitochondrial-associated gene networks shaped by nutrient timing.

## Highlights

• TRF reorganizes mitochondrial-associated gene rhythms into a cross-tissue network
• TRF nearly quadruples globally aligned mitochondrial-associated transcripts
• Mitochondria integrate nutrient timing across organ systems
• Coq10b marks coordinated mitochondrial rhythmicity across tissues

## Graphical abstract

**Figure:**
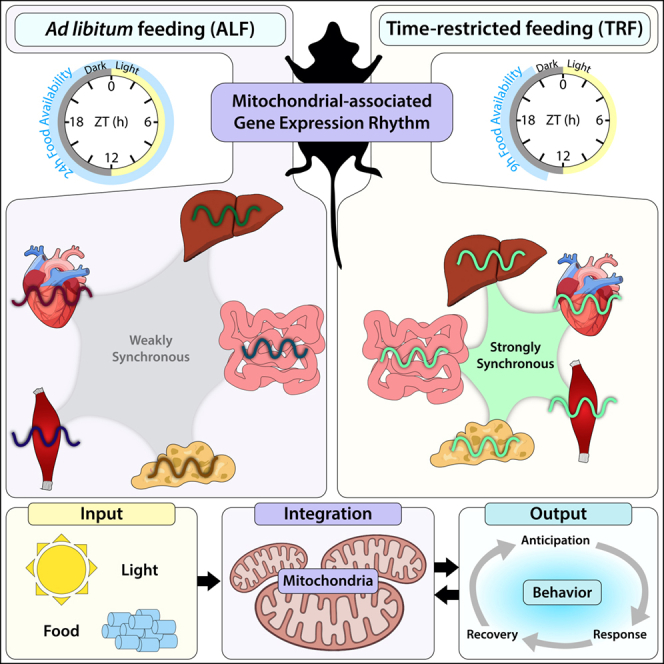

## Introduction

Organisms rely on circadian clocks to process environmental information, anticipate daily challenges, and adapt their physiology to optimize behavior and energy use.^1^^,^^2^^,^^3^^,^^4^^,^^5^^,^^6^^,^^7^^,^^8^^,^^9^^,^^10^ These clocks coordinate transcriptional and metabolic programs across tissues, aligning physiology with time-of-day and nutrient availability.^11^^,^^12^^,^^13^^,^^14^^,^^15^^,^^16^^,^^17^^,^^18^^,^^19^^,^^20^^,^^21^ Among the key downstream effectors of these rhythms are mitochondria—the cell’s biochemical processors^22^—whose function oscillates over the 24 h cycle to match cellular energy demand and redox state.^23^^,^^24^^,^^25^^,^^26^ However, while circadian and metabolic rhythms have been extensively characterized within individual tissues, how mitochondrial-associated transcriptional programs are temporally coordinated across the body remains poorly understood.

Time-restricted feeding (TRF)—restricting food access to the active phase without reducing total caloric intake—has been shown to reinforce circadian rhythmicity in peripheral tissues and improve metabolic health.^12^^,^^13^^,^^16^ TRF reorganizes daily oscillations in gene expression, metabolite levels, and energy utilization, often reversing dampened or misaligned circadian rhythms associated with *ad libitum* feeding (ALF) or high-fat diets (HFDs).^11^^,^^12^^,^^18^^,^^19^^,^^21^ Yet, despite extensive characterization of TRF’s effects within single tissues, whether and how TRF influences the temporal organization of mitochondrial-associated transcripts (MATs) across multiple organs remains unknown.

Mitochondria are central effectors of metabolic homeostasis and are increasingly recognized as rhythmically regulated organelles.^22^^,^^23^^,^^24^^,^^25^^,^^26^^,^^27^ Across multiple tissues, mitochondrial processes such as oxidative phosphorylation, substrate utilization, and redox balance display daily oscillations, driven by both intrinsic circadian clocks and systemic cues. Transcriptomic and proteomic studies have identified the rhythmic expression of mitochondrial-associated genes within individual tissues, with proteomic analyses revealing robust daily oscillations in mitochondrial enzymes and metabolic pathways.^27^^,^^28^^,^^29^ However, most studies to date have examined mitochondrial rhythms in isolation—focusing on single organs or cell types—leaving unresolved whether mitochondrial-associated transcriptional programs are temporally coordinated across tissues at the systems level.

Feeding-fasting cycles are among the strongest systemic signals influencing circadian physiology, providing temporal information that complements the light-driven entrainment of the central clock.^11^^,^^12^^,^^13^^,^^14^^,^^16^^,^^21^^,^^30^^,^^31^ TRF, which confines food intake to a consistent daily window without altering caloric intake, has been shown to reinforce circadian rhythms in peripheral tissues and improve metabolic outcomes across diverse physiological contexts.^5^^,^^11^^,^^13^^,^^18^^,^^19^^,^^32^ Importantly, TRF does not necessarily increase the amplitude of individual molecular rhythms uniformly across tissues; rather, it may influence circadian physiology by aligning temporal programs across organs through coordinated metabolic and hormonal cues. Despite growing interest in TRF as a modulator of circadian physiology, whether nutrient timing influences the temporal coordination of mitochondrial-associated transcriptional programs across tissues remains unknown.

Here, we tested the hypothesis that nutrient timing influences the cross-tissue temporal organization of MAT expression. Leveraging a high-resolution, 22-tissue circadian transcriptomic dataset collected under ALF or TRF conditions,^14^ we developed an analytical framework to quantify the temporal coordination of MATs both within and across tissues. Rather than assessing rhythmicity within individual organs alone, our approach focuses on the degree to which temporal expression profiles are aligned across the organism, capturing systems-level patterns of transcriptional coordination. Using correlation- and phase-based metrics, we identify MATs that exhibit coordinated temporal dynamics across multiple tissues and examine how these patterns are reshaped by feeding time. Together, this study defines a previously unexplored dimension of circadian regulation—cross-tissue temporal coordination of mitochondrial-associated transcription—and establishes a descriptive framework for investigating how nutrient timing organizes mitochondrial gene networks at the whole-body level.

## Results

### Analytical framework for assessing mitochondrial-associated transcript temporal coordination across tissues

**Figure 1:**
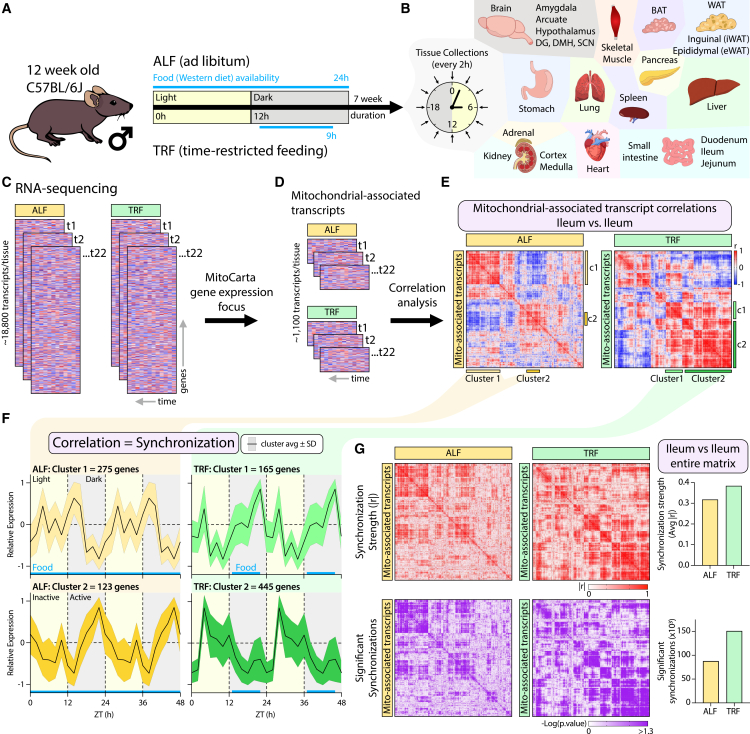
Analytical framework for assessing mitochondrial-associated transcript temporal coordination across tissues (A) Overview of experimental design adapted from Deota S. et al.^14^ (B) Twenty-two tissues were collected every 2 h over a 24 h cycle from mice (*n* = 2/time point) maintained under ad libitum-fed (ALF) or time-restricted-fed (TRF) mice. (C) Organization of RNA-sequencing data across tissues and feeding conditions. (D) Filtering of transcripts to those annotated in the Mouse MitoCarta3.0 database. (E) Representative correlation matrices of MitoCarta3.0-annotated circadian gene expression in ileum under ALF and TRF groups, with example correlation clusters highlighted. (F) Temporal expression patterns of all genes within example clusters shown in (E); black trace denotes cluster mean ± SD. Food availability is indicated along the x axis. (G) Representative matrices illustrate transcript synchronization strength (top, red) and the number of significant synchronizations (bottom, purple), with corresponding whole-matrix quantification. DG, dentate gyrus; DMH, dorsomedial hypothalamus; SCN, suprachiasmatic nucleus; BAT, brown adipose tissue; WAT, white adipose tissue.

To investigate how TRF influences the temporal organization of mitochondrial-associated transcription across the body, we analyzed a 22-tissue circadian RNA-seq dataset generated by Deota S. et al*.*^14^ This dataset comprises male C57BL/6J mice maintained under either ALF or TRF conditions. In the TRF paradigm, mice were fed beginning 1h into the dark phase with food access restricted to a 9 h window (Figure 1A). After 7 weeks on these regimens, tissues were collected every 2 h over a 24 h cycle (Figure 1B). RNA-sequencing yielded expression profiles for approximately 18,800 transcripts per tissue and time point across both dietary conditions (Figure 1C). To focus specifically on mitochondrial transcriptional dynamics, we filtered transcripts using the Mouse MitoCarta3.0 gene list,^33^ generating a curated set of MATs for downstream analysis (Figure 1D). Rather than assessing rhythmicity at the level of individual genes or tissues, we sought to quantify the degree to which temporal expression patterns of MATs were aligned within and across tissues. To this end, we applied pairwise correlation analysis across the 24 h time series to compare expression trajectories between MATs. Hierarchical clustering was used to group MATs with similar temporal expression profiles, enabling the visualization of coordinated transcriptional patterns within each tissue (Figure 1E). Within these clusters, correlation strength reflects the similarity of temporal expression dynamics rather than absolute expression levels (Figure 1F). To quantify temporal coordination, we calculated two complementary metrics across each tissue: (1) the mean absolute Pearson’s correlation coefficient (|r|), representing the overall strength of temporal alignment among MATs and (2) the total number of significantly correlated MAT pairs, reflecting the extent of coordinated transcriptional relationships (Figure 1G). Absolute correlation values were used in matrix-wide analyses to capture the strength of rhythmic connectivity independent of phase direction, allowing both in-phase and anti-phase temporal relationships to contribute to estimates of cross-tissue temporal coordination. In contrast, global synchronization scores described in subsequent analyses were calculated using signed correlation coefficients to specifically quantify in-phase temporal alignment across tissues, reflecting a distinct analytical objective from matrix-wide connectivity strength. Together, this analytical framework provides a quantitative and comparative approach for assessing how the feeding schedule influences the temporal coordination of mitochondrial-associated transcriptional programs across tissues, without presupposing functional coupling or direct regulatory interactions.

### Time-restricted feeding differentially alters the intra-tissue temporal coordination of mitochondrial-associated transcripts

**Figure 2:**
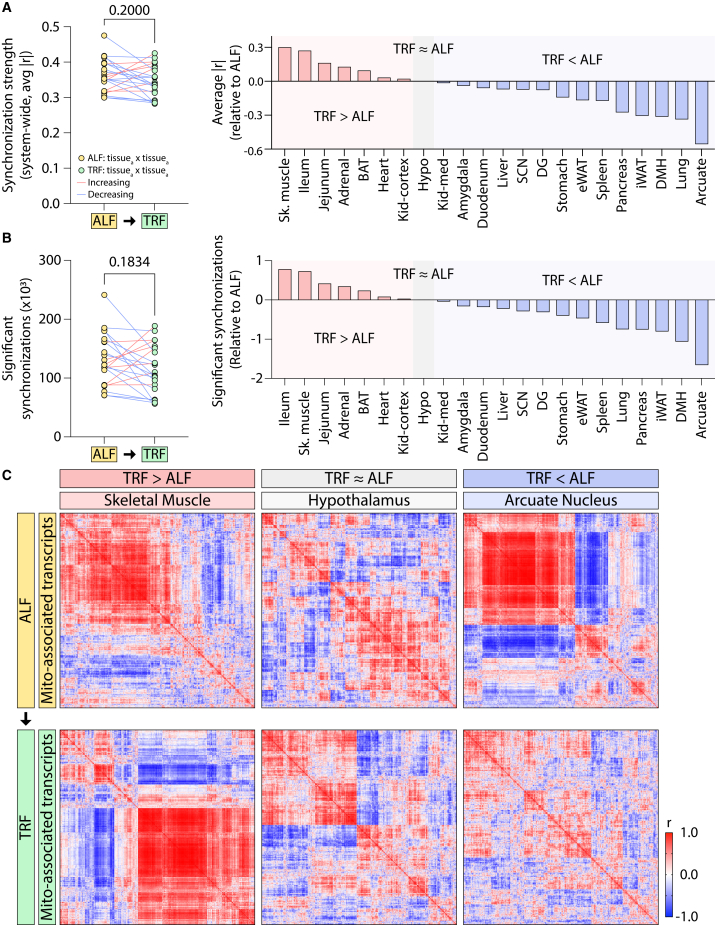
Time-restricted feeding differentially alters the intra-tissue temporal coordination of mitochondrial-associated transcripts (A) System-wide analysis of mitochondrial-associated transcript (MAT) synchronization strength across 22 tissues. Each circle represents a tissue under ALF or TRF; lines connect the same tissue across diets. Increases in transcript synchronization strength under TRF are shown in red and decreases in blue. Right panel summarizes the matrix-averaged synchronization strength per tissue, categorized as increased (red), unchanged (gray), or decreased (blue). (B) System-wide comparison of the number of significantly synchronized MATs between ALF and TRF; plot design and color-coding match (A). (C) Representative correlation matrices illustrate tissues exhibiting increased (left), unchanged (middle), or decreased (right) intra-tissue MAT synchronization between feeding conditions. Statistical significance was assessed using unpaired two-tailed t-tests; *p* values are reported numerically.

Analysis of intra-tissue MAT temporal coordination across 22 tissues revealed no global increase under TRF compared to ALF. (Figure 2A, left). Instead, individual tissues exhibited heterogeneous and diet-specific responses (Figure 2A, right). Coordination strength increased in skeletal muscle, ileum, jejunum, adrenal gland, brown adipose tissue (BAT), heart, and kidney cortex; remained largely unchanged in the hypothalamus; and decreased in the kidney medulla, amygdala, duodenum, liver, suprachiasmatic nucleus (SCN), dentate gyrus (DG), stomach, epididymal white adipose tissue (eWAT), spleen, pancreas, inguinal WAT (iWAT), dorsomedial hypothalamus (DMH), lung, and arcuate nucleus. Consistent with these patterns, the total number of significantly coordinated MAT pairs did not differ globally between dietary states (Figure 2B, left), while tissue-specific patterns closely paralleled changes in coordination strength (Figure 2B, right). Among all tissues, skeletal muscle exhibited the most pronounced increase in intra-tissue MAT coordination, whereas the arcuate nucleus showed the greatest reduction (Figure 2C, left and right). Notably, although the hypothalamus displayed minimal net change in coordination strength or number of significant MAT pairs, its internal network organization was substantially remodeled between ALF and TRF (Figure 2C, middle). Overlap analysis confirmed the extensive reorganization of coordinated MAT relationships despite similar aggregate metrics (Figure S1A), indicating that TRF can restructure intra-tissue MAT networks without uniformly altering overall coordination levels. Together, these results indicate that TRF does not uniformly strengthen MAT coordination within individual tissues, prompting us to investigate whether nutrient timing coordinates MAT expression across tissues.

### Time-restricted feeding increases the cross-tissue temporal coordination of mitochondrial-associated transcripts

**Figure 3:**
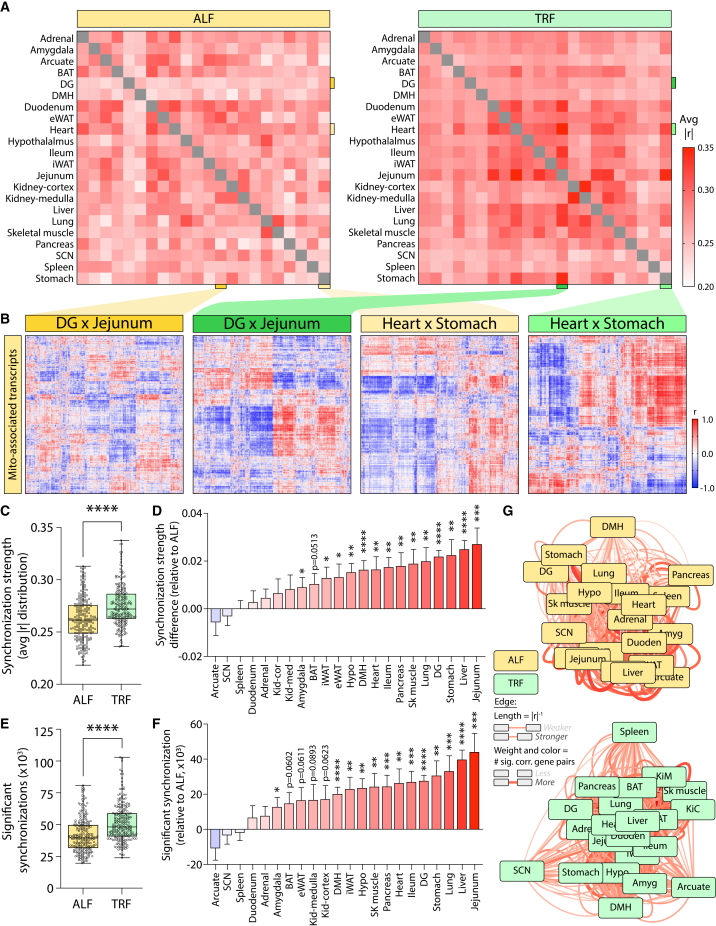
Time-restricted feeding increases the cross-tissue temporal coordination of mitochondrial-associated transcripts (A) Heatmap shows average inter-tissue MAT synchronization strength (|r|) across all tissue pairs under ALF and TRF. (B) Representative tissue-pair comparisons illustrate increased cross-tissue MAT synchronization under TRF. (C) System-wide comparison of mean inter-tissue MAT synchronization strength between dietary states. (D) Tissue-level relative change in synchronization strength from ALF to TRF; data represented as mean ± SEM. (E) System-wide comparison of the number of significantly synchronized inter-tissue MATs. (F) Tissue-level relative change in significant synchronizations from ALF to TRF. (G) Network representation of inter-tissue MAT synchronization, where edge length reflects correlation strength, and edge thickness and red intensity represent the number of significant synchronizations. DG, dentate gyrus; DMH, dorsomedial hypothalamus; SCN, suprachiasmatic nucleus; BAT, brown adipose tissue; iWAT, inguinal white adipose tissue; eWAT, epididymal white adipose tissue; Hypo, hypothalamus; KiM, kidney medulla; KiC, kidney cortex; Sk muscle, skeletal muscle; Amyg, amygdala; Arcuate, arcuate nucleus; Duoden, duodenum. Statistical significance was determined using unpaired two-tailed t-tests and is denoted as ∗, ∗∗, ∗∗∗, and ∗∗∗∗ for *p* < 0.05, 0.01, 0.001, and 0.0001, respectively.

Given the lack of a uniform increase in intra-tissue MAT coordination, we next asked whether TRF may enhance coordination across tissues. Pairwise correlation analysis of MAT temporal expression revealed a net increase in inter-tissue MAT coordination under TRF compared to ALF (Figure 3A). Representative tissue-pair comparisons—DG versus jejunum and heart versus stomach—illustrate this effect, with TRF samples (green) exhibiting stronger and more structured correlation patterns than ALF (Figure 3B). Full inter-tissue synchronization summaries, showing the summed correlation strength (Σ|r|) of each tissue across all tissue pairs under ALF and TRF, are provided in Figures S2 and S3. Across all tissue-pair comparisons, TRF generally increased inter-tissue coordination, with the exception of the arcuate nucleus, SCN, and spleen (Figures 3C and 3D). A similar trend was observed for the number of significantly coordinated MATs, with minimal changes in tissue rank order between dietary states (Figures 3E and 3F). Network analysis further highlighted a large-scale reorganization of cross-tissue connectivity: Under ALF, the liver occupied a relatively peripheral network position, whereas under TRF, it became a central hub of MAT coordination. In contrast, the SCN, arcuate nucleus, and spleen shifted toward the network periphery (Figure 3G). Together, these findings indicate that TRF enhances cross-tissue coordination of MATs, suggesting that rhythmic nutrient availability reorganizes mitochondrial-associated transcriptional networks to reinforce temporal coherence across organs. These observations raised the possibility that TRF does not merely increase pairwise tissue coordination but additionally promotes a subset of MATs that are temporally aligned across many or all tissues.

### Time-restricted feeding increases the number and diversity of globally coordinated mitochondrial-associated transcripts

**Figure 4:**
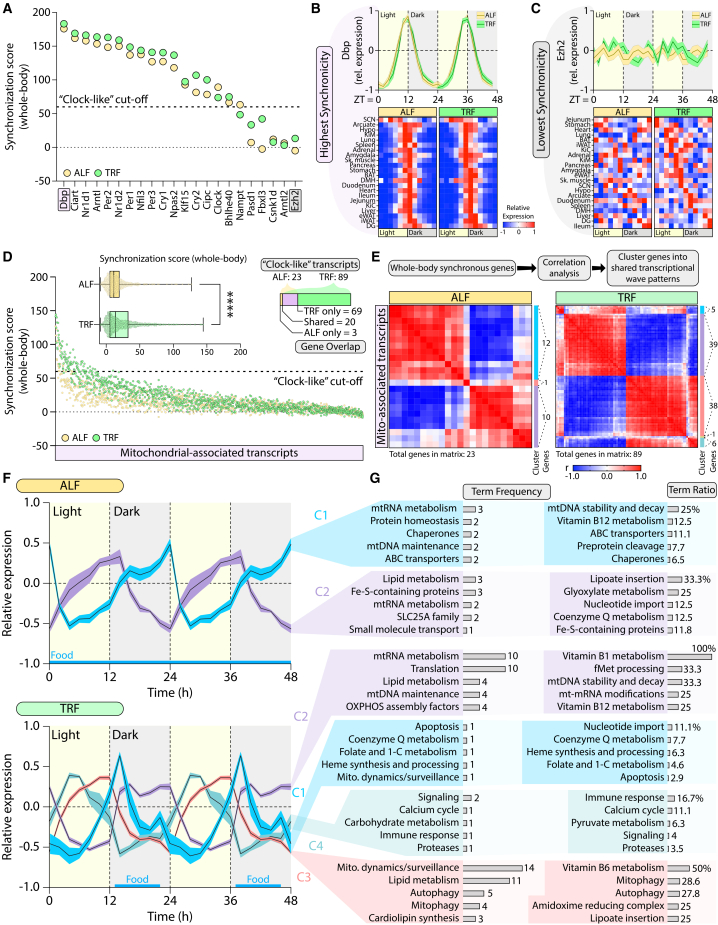
Time-restricted feeding increases the number and diversity of globally coordinated mitochondrial-associated transcripts (A) Whole-body synchronization score distribution for circadian clock genes. The dotted line indicates the first major drop-off in ALF synchronization scores and defines the “clock-like” threshold. (B) Representative example of a highly synchronized circadian gene (*Dbp*): whole-body expression waveform (top) and tissue-level profiles (bottom). (C) Representative example of a weakly synchronized circadian gene (*Ezh2*), displayed as in (B). (D) Distribution of whole-body synchronization scores for all MATs under ALF and TRF, shown as collapsed (top) and expanded (bottom) views. Top-right inset shows the overlap of MATs exceeding the “clock-like” threshold under both conditions. (E) Correlation matrices of all globally synchronous MATs under ALF and TRF, with cluster assignments and gene counts indicated. (F) Temporal expression patterns of MAT clusters. (G) Top five MitoCarta3.0 pathway-associated terms ranked by frequency (absolute count) and ratio (cluster count/total possible) for each globally synchronous MAT cluster. Gene expression patterns are shown over two consecutive days as cluster mean ± SEM. Statistical significance was determined using unpaired two-tailed t-tests and is denoted as ∗, ∗∗, ∗∗∗, and ∗∗∗∗ for *p* < 0.05, 0.01, 0.001, and 0.0001, respectively. Data presented in B, C, and F are represented as mean ± SEM.

Given the widespread increase in inter-tissue coordination under TRF, we next asked whether specific MATs exhibit coordinated temporal expression across many tissues simultaneously, and how this property is influenced by feeding behavior. To quantify this, we developed a whole-body synchronization score defined as the sum of pairwise correlation coefficients for each MAT across all tissue pairs. Correlation signs were preserved (rather than using |r|) to avoid inflating anti-phased relationships and to capture the directionality of temporal alignment. To benchmark this metric, we first analyzed canonical circadian genes (Figure 4A). As expected, many core clock components exhibited high levels of synchronization across tissues. *Dbp* showed the most tightly aligned rhythmic expression across all 22 tissues in both ALF and TRF conditions (Figure 4B), whereas *Ezh2* ranked the lowest and lacked cross-tissue coherence (Figure 4C). The first pronounced decline in synchronization score among benchmark genes occurred between *Nampt* and *Pasd1* (∼y = 60), which we operationally defined as a “clock-like” threshold for global temporal coordination (Figure 4A).

Applying this framework to all MATs revealed a global upward shift in synchronization scores under TRF (Figure 4D, bottom). Across all MATs, average synchronization was higher under TRF than ALF (Figure 4D, top left), and the number of globally coordinated transcripts exceeding the clock-like threshold increased from 23 in ALF to 89 in TRF (Figure 4D, top right). Overlap analysis showed that 20 MATs were shared between dietary states, 3 were unique to ALF, and 69 were unique to TRF. A complete list of MATs and their synchronization scores is provided in Table S1. Hierarchical clustering of globally synchronous MATs revealed two dominant transcriptional waves in ALF and four under TRF (Figure 4E). In ALF, the two clusters oscillated in near opposition, aligning with light-dark and dark-light transitions (Figure 4F, top). In contrast, TRF produced a more complex temporal architecture consisting of four distinct waves corresponding to successive feeding-fasting phases across the dark and light cycles (Figure 4F, bottom). Full gene lists for each cluster, ranked by synchronization score, are provided in Figure S4.

Functional annotation using MitoCarta3.0 categories revealed distinct pathway enrichments associated with each wave (Figure 4G). Under ALF, wave C1 was enriched for mtRNA metabolism and mtDNA maintenance, while wave C2 was dominated by lipid metabolism, Fe-S cluster assembly, lipoate insertion, and glyoxylate pathways. Under TRF, wave C1 was linked to nucleotide import, coenzyme Q metabolism, and heme synthesis; wave C2 to mtRNA processing, translation, and vitamin B1 metabolism; wave C3 to mitochondrial dynamics, lipid metabolism, vitamin B6 metabolism, mitophagy, and autophagy; and wave C4 to signaling, immune, and calcium pathways. Together, these findings demonstrate that TRF increases both the extent and organizational complexity of globally coordinated MAT expression, revealing a system-wide restructuring of mitochondrial temporal programs.

To explore potential transcriptional contexts associated with these global MAT waveforms, we performed transcription factor (TF) enrichment analysis for each wave using ChEA3.^34^ This analysis identified partially overlapping sets of TFs associated with MAT waves under both ALF and TRF conditions, with no single TF accounting for all transcripts within a given waveform (Figure S5). Instead, individual waves were characterized by combinatorial TF associations, consistent with distributed transcriptional influences underlying coordinated temporal expression. Given the expansion and diversification of globally synchronized MATs under TRF, we next asked whether specific transcripts occupy particularly central or informative positions within this coordinated transcriptional network. Among the top-ranked globally synchronized MATs, *Coq10b* emerged as a prominent candidate based on its strong whole-body synchrony and established role in mitochondrial electron transport and coenzyme Q biology.

### Coenzyme Q10B (*Coq10b*) is a highly temporally coordinated mitochondrial-associated transcript under time-restricted feeding

**Figure 5:**
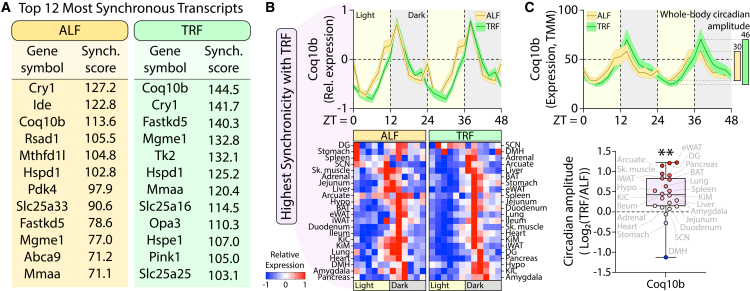
Coenzyme Q10B (*Coq10b*) is a highly temporally coordinated mitochondrial-associated transcript under time-restricted feeding (A) Top 12 MATs with the highest synchronization scores under ALF and TRF. (B) Whole-body expression waveform (top) and tissue-level expression profiles (bottom) of *Coq10b*, the most synchronized MAT under TRF. Expression is shown over two consecutive days as mean ± SEM. (C) TMM-normalized *Coq10b* expression waveforms under ALF and TRF (top), with corresponding whole-body circadian amplitude (right). Bottom panel shows relative change in circadian amplitude (TRF/ALF) across tissues; each tissue is indicated in gray. Statistical significance was determined using a one-sample *t* test and is denoted as ∗, ∗∗, ∗∗∗, and ∗∗∗∗ for *p* < 0.05, 0.01, 0.001, and 0.0001, respectively. Data presented in B and C are represented as mean ± SEM.

Having established that TRF enhances global MAT synchrony, we next identified the most highly synchronized MATs in each dietary condition (Figure 5A). Under the ALF state, *Cry1, Ide, Coq10b, Rsad1, Mthfd1l, Hspd1, Pdk4, Slc25a33, Fastkd5, Mgme1, Abca9,* and *Mmaa* exhibited the highest whole-body synchronization scores. In contrast, under TRF, the top-ranked transcripts included *Coq10b, Cry1, Fastkd5, Mgme1, Tk2, Hspd1, Mmaa, Slc25a16, Opa3, Hspe1, Pink1,* and *Slc25a25*. Notably, *Coq10b*—encoding coenzyme Q10B—rose from third place under ALF to the most globally synchronized transcript across all 22 tissues under TRF, surpassing even *Cry1*, a core component of the circadian transcription-translation feedback loop. At the whole-body level, *Coq10b* expression followed a similar temporal pattern under ALF and TRF, but exhibited markedly reduced variability in the TRF state (Figure 5B, top). The examination of the individual tissue-specific expression waves, organized by peak phase, revealed near-complete temporal alignment across tissues, except for the SCN and DMH, which retained distinct expression profiles (Figure 5B, bottom).

To assess whether enhanced synchrony was accompanied by changes in rhythmic amplitude, we compared absolute whole-body *Coq10b* expression profiles between dietary states (Figure 5C, top). TRF significantly increased circadian amplitude—from 30 in ALF to 46 in TRF—reflecting elevated peak expression during the dark (active) phase and reduced trough expression during the light (inactive) phase. At the tissue level, most organs exhibited increased *Coq10b* amplitude under TRF (Figure 5C, bottom), with the strongest responses observed in eWAT, DG, arcuate nucleus, skeletal muscle, and pancreas. In contrast, the SCN, stomach, and DMH displayed reduced amplitudes. Together, these findings identify *Coq10b* as a prominent and highly synchronized MAT under TRF, positioning it as a candidate integrator of mitochondrial rhythmicity and systemic metabolic timing. The emergence of *Coq10b* as the most globally synchronized MAT under TRF prompted us to use it as a molecular entry point to investigate how nutrient timing and circadian cues jointly organize mitochondrial-associated transcription across tissues.

### Nutrient timing and circadian cues jointly shape mitochondrial-associated transcript rhythmicity

**Figure 6:**
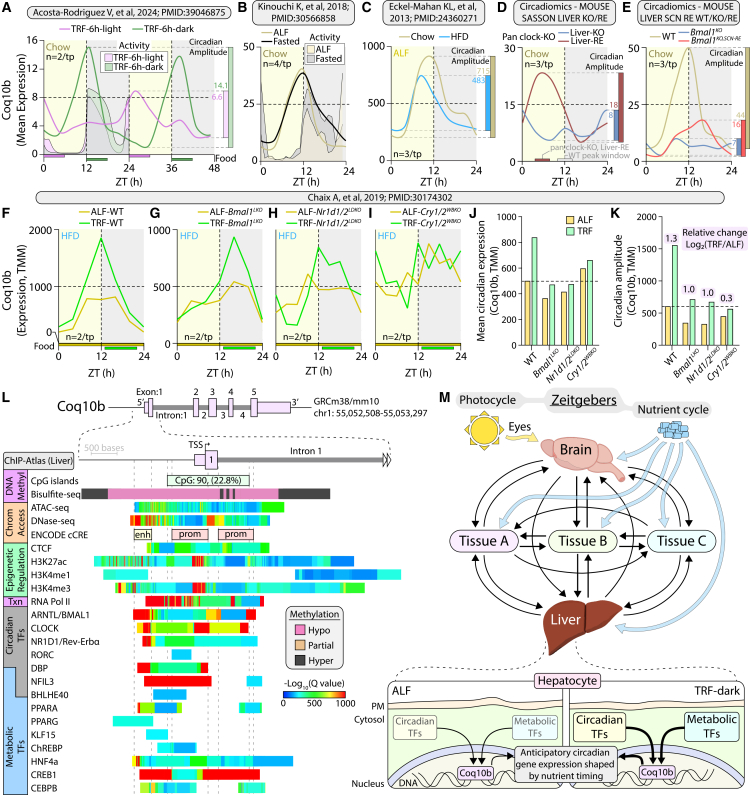
Nutrient timing and circadian cues jointly shape mitochondrial-associated transcript rhythmicity All data depict *Coq10b* expression in the mouse liver. Feeding condition is indicated in the upper left of each panel, with feeding time shown along the x axis where applicable. Circadian expression amplitude is shown to the right of each plot, and sample size per time point is indicated on the left. Locomotor activity is underlaid (A and B). Dataset source or reference is listed above each panel. (A) Mice fed an isocaloric diet restricted to either the light or dark phase. (B) Mice fed ALF or fasted for 24 h prior to tissue collection. (C) Mice fed a standard chow or high-fat diet (HFD) under ALF conditions. (D) Pan clock-KO (*Bmal1*^*KO*^) mice lacking *Bmal1* expression (Liver-KO) or with liver-specific *Bmal1* re-expressed (Liver-RE). Peak *Coq10b* expression times are indicated along the x axis. (E) Pan clock-KO mice with *Bmal1* (*Bmal1*^KO^) re-expression restricted to the suprachiasmatic nucleus (SCN; *Bmal1*^KO,SCN−RE^), or wild-type controls (WT). (F) Wild-type mice under ALF (ALF-WT) or TRF (TRF-WT). (G) Liver-specific *Bmal1*-KO mice fed ALF (ALF-*Bmal1*^LKO^) or TRF (TRF-*Bmal1*^LKO^). (H) Liver-specific *Nr1d1/2* double KO mice under ALF (ALF-*Nr1d1/2*^LDKO^) or TRF (TRF-*Nr1d1/2*^LDKO^). (I) Whole-body *Cry1/2* double KO mice under ALF (ALF-*Cry1/2*^WBKO^) or TRF (TRF- *Cry1/2*^WBKO^). (J) Mean circadian expression levels and (K) circadian amplitudes of *Coq10b* across models in (F–I). Relative change in circadian expression [Log_2_(TRF/ALF)] is shown above each genotype. (L) Meta-analysis of public data from the UCSC Genome Browser (GRCm38/mm10) and ChIP-Atlas (liver datasets only) shows epigenomic features and transcription factor binding within ±2 kb of the *Coq10b* transcription start site (TSS). (M) Schematic model summarizes the proposed regulatory context of *Coq10b* and its association with the circadian coordination of mitochondrial-associated gene expression under ALF and TRF-dark conditions. Arrows from *Coq10b* indicate inferred transcriptional associations and regulatory context rather than direct causal or physiological relationships. *Coq10b* is depicted as a representative transcript embedded within coordinated circadian and metabolic regulatory networks shaped by nutrient timing.

Having identified *Coq10b* as the most globally synchronous MAT under TRF, we next used its hepatic expression as a molecular readout to explore factors associated with coordinated MAT rhythmicity. The liver was selected due to its central role in metabolic homeostasis, its hub-like position in our MAT network (Figure 3G), and the abundance of publicly available circadian transcriptomic datasets. To contextualize *Coq10b* regulation across diverse metabolic and circadian perturbations, we analyzed multiple independent liver transcriptomic datasets (summarized in Table S2). We first asked whether the *Coq10b* expression wave aligned more closely with the light-dark cycle, feeding schedule, or locomotor activity. The analysis of liver transcriptomes from male mice fed an isocaloric diet restricted to either the light or dark phase (TRF-6h-light vs. TRF-6h-dark)^21^ revealed that *Coq10b* expression primarily tracked with food availability (Figure 6A). Despite equivalent feeding durations, the TRF-6h-light mice failed to achieve the same circadian amplitude as TRF-6h-dark mice, suggesting limits in transcriptional amplitude remodeling when feeding occurs at an atypical circadian phase.

To determine whether *Coq10b* expression reflects an acute fasting response or a learned rhythmic program, we analyzed data from Kinouchi K. et al.*,*^17^ in which mice experienced a single 24 h fast. *Coq10b* expression remained stable, indicating that its rhythmic regulation is not acutely driven by fasting (Figure 6B). Because HFD feeding disrupts both metabolic and circadian rhythms,^15^^,^^35^ we next examined *Coq10b* in mice fed standard chow or HFD long-term. Consistent with metabolic dysregulation, *Coq10b* rhythmic amplitude was markedly reduced under HFD conditions (Figure 6C). Together with the TRF-induced increase in amplitude (Figure 5C), these findings suggest that *Coq10b* rhythmic expression reflects metabolic state and systemic circadian alignment.

To further assess the multi-tissue contribution to mitochondrial rhythmicity, we used publicly available datasets from the CircadiOmics web server.^36^^,^^37^ In the “MOUSE SASSON LIVER KO” and “MOUSE SASSON LIVER RE” datasets, liver *Coq10b* expression was compared across pan-clock-KO (*Bmal1*^*KO*^; Liver-KO in figure) mice and mice with liver-specific clock restoration (liver-RE). Global loss of the clock abolished *Coq10b* rhythmicity, whereas the restoration of *Bmal1* exclusively in the liver re-established oscillatory expression but failed to restore phase timing (Figure 6D). Similarly, in the “MOUSE LIVER SCN RE WT,” MOUSE LIVER SCN RE KO,” and “MOUSE LIVER SCN RE RE” datasets, reintroduction of *Bmal1* exclusively in the SCN partially restored *Coq10b* rhythmicity and amplitude (Figure 6E). Together, these findings indicate that coordinated *Coq10b* rhythmicity depends on timing cues arising from both central and peripheral oscillators.

To directly test if *Coq10b* rhythmicity requires an intact molecular clock, we analyzed liver datasets from Chaix A. et al. (2019),^12^ comparing rhythmic gene expression across ALF and TRF conditions in wild-type and clock-deficient mice. As expected, TRF enhanced *Coq10b* amplitude in wild-type livers (Figures 6F and 6K). Mice lacking hepatic *Bmal1* (*Bmal1*^*LKO*^) retained this amplitude increase despite a phase shift (Figures 6G and 6K), while those lacking hepatic *Nr1d1/2* (*Nr1d1/2*^*LDKO*^) also exhibited elevated amplitude under TRF (Figures 6H and 6K). In contrast, global *Cry1/2* double-KO mice (*Cry1/2*^*WBKO*^) showed minimal amplitude change but higher baseline expression, potentially reflecting loss of Cry-mediated transcriptional repression (Figures 6I–6K). Collectively, these findings underscore the intertwined contributions of circadian and metabolic regulatory systems in shaping *Coq10b* rhythmic expression.

To explore the regulatory architecture underlying this dual control, we examined publicly available ChIP-seq epigenomic data surrounding the *Coq10b* transcription start site (TSS, ±2 kb) using ChIP-Atlas and the UCSC Genome Browser. This region overlaps a hypomethylated CpG island (Figure 6L), exhibits strong chromatin accessibility (ATAC-seq and DNase-seq), and contains multiple ENCODE-predicted *cis*-regulatory elements, including two promoters and one enhancer. The enrichment of CTCF, H3K27ac, H3K4me1, H3K4me3, and RNA Pol II is consistent with active transcriptional regulation. Notably, binding of several circadian and metabolic TFs within this region supports an integrated regulatory framework for *Coq10b* expression.

Altogether, these results support the model summarized in Figure 6M. Light-derived temporal cues are processed by the SCN and relayed to peripheral tissues, while feeding-fasting cycles provide metabolic timing signals that act both directly and indirectly on organs such as the liver. Under ALF, circadian and metabolic transcriptional regulators jointly shape *Coq10b* rhythmic expression in the anticipation of energy demand. When TRF is aligned with the organism’s active phase, these regulatory systems act synergistically to enhance coordinated MAT rhythmicity across tissues, potentially representing a transcriptional mechanism through which nutrient timing promotes systemic metabolic efficiency and resilience.

## Discussion

In this study, we sought to understand how mitochondria process and adapt to environmental information, such as nutrient timing, to promote whole-body metabolic health. Leveraging a comprehensive, multi-tissue circadian transcriptomic dataset^14^ from mice maintained under ALF or TRF, we focused on the mitochondrial-associated transcriptome—genes annotated in the MitoCarta3.0 that encode mitochondrial-localized proteins. This systems-level approach revealed that TRF induces widespread reorganization of MAT rhythmicity across tissues, altering intra-tissue coherence while simultaneously enhancing the cross-tissue alignment of rhythmic expression. Together, these findings suggest that TRF coordinates mitochondrial-associated transcriptional dynamics as an integrated network spanning tissues rather than as independent oscillators confined to individual organs.

TRF modulated rhythmic mitochondrial-associated gene expression both within and across tissues, acting as a temporal unifier of metabolic programs. Consistent with prior work showing that TRF restores circadian rhythmicity in peripheral clocks and metabolic pathways, our results extend this principle to mitochondrial-associated gene networks. Rather than uniformly increasing coordination strength in all tissues, TRF reshaped the structure and timing relationships of MAT expression, resulting in greater alignment of rhythmic phases across organs. This coordinated temporal organization likely reflects improved systemic alignment of energy metabolism—synchronizing nutrient oxidation, redox homeostasis, and biosynthetic processes across organs. Such multi-tissue coordination of MATs may represent a molecular basis through which TRF confers its well-established metabolic benefits.

Mechanistically, the observed coordination of MAT rhythmicity under TRF likely arises from bidirectional interactions between nutrient-responsive signaling and circadian transcriptional control. Feeding-fasting cycles regulate key metabolic cofactors such as NAD^+^/NADH, AMP/ATP, and acetyl-CoA, which modulate the activity of sirtuins, AMPK, and acetyltransferases.^23^^,^^24^^,^^25^^,^^26^^,^^27^ These metabolic sensors, in turn, interface with circadian TFs (BMAL1, CLOCK, PER, CRY) and co-regulators (PGC1α, NRF1/2, SIRT1) to shape rhythmic mitochondrial-associated gene expression.^9^^,^^15^^,^^22^^,^^26^ The persistence of rhythmic MAT expression even in tissues with diminished clock strength supports the idea that metabolic oscillations can contribute to maintaining coordinated mitochondrial-associated transcriptional programs. Thus, mitochondria may act as integrators of nutrient timing, translating systemic metabolic cues into coherent, tissue-spanning temporal expression patterns.

Our results expand upon previous “Circadiomic” studies that emphasized the tissue specificity of rhythmic gene expression.^2^^,^^3^^,^^4^^,^^5^^,^^6^^,^^7^^,^^9^^,^^10^^,^^11^^,^^12^^,^^16^^,^^20^^,^^26^ In contrast, we reveal an additional layer of cross-tissue coordination that is strengthened under TRF. By focusing on the mitochondrial-associated transcriptome, we uncovered a subset of MATs that exhibit highly aligned rhythmic timing across multiple tissues, suggesting the existence of a shared temporal framework for MAT regulation. These globally rhythmic transcripts may function as network hubs that couple local mitochondrial processes to organismal metabolic time.

To illustrate this systemic coordination, we examined *Coq10b*, which emerged as the most strongly cross-tissue-aligned MAT under TRF. *Coq10b* encodes a coenzyme Q-binding protein essential for electron transport chain efficiency and redox regulation^38^^,^^39^—core processes underlying metabolic health. Its robust amplitude and consistent phase alignment across tissues suggest that *Coq10b* may serve as a molecular indicator of coordinated MAT rhythmicity. Analyses of publicly available datasets showed that *Coq10b* rhythmicity tracks feeding cycles, becoming enhanced under metabolically favorable conditions such as TRF and attenuated under metabolic stress (e.g., HFDs). Moreover, genetic disruption of the circadian clock in the liver or SCN revealed that *Coq10b* rhythmicity depends on both central and peripheral timing systems, implying multi-level regulatory control rather than reliance on a single tissue oscillator. Epigenomic and TF binding analyses further uncovered dual circadian and metabolic regulatory inputs at the *Coq10b* promoter, supporting its role as a convergence point for these two information streams. Together, these findings position *Coq10b* as a model for how mitochondria integrate nutrient timing and circadian control to achieve coordinated rhythmic regulation across tissues.

In summary, this work reveals that TRF reorganizes the mitochondrial-associated transcriptome into a coordinated, nutrient-entrained, multi-tissue network. By uncovering this tissue-spanning temporal alignment of MATs, we provide a framework for understanding how nutrient timing engages mitochondria as systemic integrators of metabolic information. These findings bridge circadian biology and metabolic regulation, offering insight into how temporal nutrition strategies may enhance mitochondrial efficiency and promote whole-body metabolic resilience.

### Limitations of the study

While our analysis provides transcript-level evidence for the global coordination of MATs, several limitations remain. These findings describe transcript-level temporal organization and do not directly assess mitochondrial function or metabolic output. Whether these rhythmic transcriptional programs translate into oscillations in protein abundance, enzymatic activity, or mitochondrial physiology remains to be determined. Bulk-tissue analyses may also obscure cell-type-specific differences in mitochondrial timing. Future integration of single-cell temporal transcriptomics, mitochondrial proteomics, and metabolomics will be essential to map how TRF organizes mitochondrial function at cellular and organellar scales. Experimental manipulation of tissue-specific and systemic clocks will help disentangle whether cross-tissue MAT coordination arises from direct transcriptional coupling or from endocrine and metabolic feedback loops. Finally, all data were obtained from male C57BL/6J mice at a single young adult age range; extending these analyses to female mice, other genetic backgrounds, and additional life stages will be important to determine the generality of these temporal coordination patterns.

## Resource availability

### Lead contact

Further information and requests for resources should be directed to and will be fulfilled by the Lead contact, Dylan C. Sarver (dsarver@g.ucla.edu).

### Materials availability

This study did not generate new unique reagents or materials.

### Data and code availability

Original code utilized for cluster-specific mitochondrial-associated gene expression pathway analysis has been deposited at Figshare (https://doi.org/10.6084/m9.figshare.30594017) and is publicly available as of the date of publication. The repository contains custom scripts, associated data tables, and documentation demonstrating their use for pathway analysis, representing the primary custom computational component of this study. All analyses were performed in R (version 4.3.2) using packages listed in the key resources table or GraphPad Prism (version 10.6.1). A record of relevant package versions and dependencies is provided within the repository. Any additional information required to reanalyze the data reported in this article is available from the lead contact upon request.

## Acknowledgments

We thank the investigators at the Salk Institute, University of California San Diego, University of California Irvine, and University of Texas Southwestern Medical Center who generated the circadian RNA-seq datasets used in this study. We also acknowledge the developers and curators of the MitoCarta3.0, UCSC Genome browser, ChIP-Atlas, and CircadiOmics web portal for providing accessible, well-curated, and publicly available resources that enabled these analyses. This work was supported by the 10.13039/100000002National Institutes of Health, 10.13039/100000050National Heart, Lung, and Blood Institute - T32 HL144449 and U54HL170326, and the 10.13039/100000002National Institutes of Health, 10.13039/100000062National Institute of Diabetes and Digestive and Kidney Diseases – R01DK117850.

## Author contributions

Conceptualization, D.C.S.; data curation, D.C.S. and Y.M.; formal analysis, D.C.S.; funding acquisition, D.C.S. and A.J.L.; investigation, D.C.S.; methodology, D.C.S.; project administration, D.C.S.; resources, D.C.S. and A.J.L; software, D.C.S.; supervision, D.C.S., A.J.L., and C.S.C.; visualization, D.C.S.; writing – original draft, D.C.S.; writing – review and editing, D.C.S., A.J.L., C.S.C., and Y.M.

## Declaration of interests

The authors declare no competing interests.

## STAR★Methods

### Key resources table

**Table:** 

REAGENT or RESOURCE	SOURCE	IDENTIFIER
**Deposited data**		

*Ad libitum* feeding vs. TRF-9h-Dark – 22 tissue mouse RNA-seq	Deota et al.^14^	GSE190389
Isocaloric TRF-6h-Light vs. TRF-6h-Dark – mouse liver RNA-seq	Acosta-Rodriguez et al.^21^	GSE266543
*Ad libitum* feeding vs. 24 h fasted – mouse liver RNA-seq	Kinouchi et al.^17^	GSE107787
Standard chow fed vs. high fat-diet fed – mouse liver RNA-seq	Eckel-Mahan et al.^15^	GSE52333
Pan-clock-KO and Liver clock restoration – mouse liver RNA-seq	CircadiOmics^36^^,^^37^^,^^40^	“MOUSE SASSON LIVER KO” and “MOUSE SASSON LIVER RE” https://circadiomics.igb.uci.edu
Pan-clock-KO and SCN clock restoration – mouse liver RNA-seq	CircadiOmics^36^^,^^37^^,^^40^	“MOUSE LIVER SCN RE WT”, “MOUSE LIVER SCN RE KO”, and “MOUSE LIVER SCN RE RE” https://circadiomics.igb.uci.edu
ALF vs. TRF-9h-Dark – mouse liver RNA-seq	Chaix et al.^12^	GSE102072
ALF-Bmal1-LKO vs. TRF-9h-Dark-Bmal1-LKO – mouse liver RNA-seq	Chaix et al.^12^	GSE102072
ALF-Nr1d1/2-LDKO vs. TRF-9h-Dark-Nr1d1/2-LDKO – mouse liver RNA-seq	Chaix et al.^12^	GSE102072
ALF-Cry1/2-WBKO vs. TRF-9h-Dark-Cry1/2-WBKO – mouse liver RNA-seq	Chaix et al.^12^	GSE102072
UCSC Genome Browser – mouse (GRCm38/mm10)	Perez et al.^41^	https://genome.ucsc.edu
MitoCarta3.0 dataset – mouse	Rath et al.^33^	https://personal.broadinstitute.org/scalvo/MitoCarta3.0/mouse.mitocarta3.0.html

**Software and algorithms**		

R v4.3.2	R Core Team^42^	https://www.R-project.org/
WGCNA	Langfelder and Horvath^43^^,^^44^	https://cran.r-project.org/web/packages/WGCNA/index.html
pheatmap	CRAN	https://cran.r-project.org/web/packages/pheatmap/index.html
MitoCarta3.0 pathway analysis by terms (code and annotation resources)	Figshare	https://doi.org/10.6084/m9.figshare.30594017
Cytoscape v3.10.2	Shannon et al.^45^	https://cytoscape.org
ChIP-Atlas	Zou et al.^46^; Zou et al.^47^; Oki et al.^48^	https://chip-atlas.org
ChEA3-ChIP-X Enrichment Analysis v3	Keenan et al.^34^	https://maayanlab.cloud/chea3/
GraphPad Prism v10.6.1	GraphPad Software	https://www.graphpad.com
Adobe Illustrator	Adobe Inc.	https://adobe.com/products/illustrator

### Method details

#### Data acquisition for multi-tissue analysis of mitochondrial-associated transcript dynamics

All transcriptomic data used in the intra-tissue, inter-tissue, and global synchronization of mitochondrial-associated transcripts (MATs) were obtained from Deota S. et al. (GEO accession GSE190389),^14^ which profiled 22 tissues collected every 2 h over a 24 h cycle from male C57BL/6J mice maintained under *ad libitum* (ALF) or time-restricted feeding (TRF, food access limited to a 9 h window during the dark phase) for 7 weeks. Animals were adults at the time of tissue collections (*n* = 2/time point) and key study parameters are highlighted in Figure 1. Full experimental details are provided in the original publication.

#### Mitochondrial-associated transcriptome selection

To focus specifically on mitochondrial regulation, we extracted genes annotated in the Mouse MitoCarta3.0,^33^^,^^49^^,^^50^ representing nuclear- and mitochondrial-encoded transcripts whose protein products localize to mitochondria. Only transcripts detected across all sampled time points within a given tissue were retained for analysis, ensuring comparability of temporal dynamics across dietary states.

#### Data preprocessing and normalization

Raw or processed expression values were downloaded from the original dataset. For each tissue, expression values represent the mean of all biological replicates collected at each circadian time point. For each tissue and feeding condition, expression values were min-max normalized per gene across time to preserve temporal patterns while minimizing differences in absolute expression amplitude between genes. This normalization was applied only for correlation-based analyses and not for amplitude-based comparisons, which used unscaled expression values as specified below.

#### Correlation and synchronization analyses

Pairwise Pearson correlation coefficients (r) were computed between all MATs within each tissue to assess intra-tissue coordination of temporal expression profiles across the 24 h cycle. Intra-tissue MAT synchronization strength was quantified as the mean absolute correlation coefficient (|r|) across each tissue’s full correlation matrix. These average |r| values were used to compare synchronization strength across tissues and to evaluate system-wide trends in coordination under ALF and TRF conditions, rather than to infer specific gene-gene regulatory relationships. Signed correlation metrics were retained for analyses focused specifically on globally in-phase rhythmic alignment, where directional phase relationships were biologically central. The difference in matrix-average |r| between TRF and ALF was used to rank tissues according to their relative synchronization response to dietary timing. Pairwise correlation *p*-values were calculated using standard R statistical functions and were interpreted as descriptive measures of association strength across the distribution of MAT correlations. Because the goal of this analysis was to characterize global coordination patterns rather than to identify individual gene-gene hypotheses, no multiple-comparison correction was applied. Intra-tissue MAT correlation matrices were visualized using hierarchical clustering to order rows and columns, with a correlation-based dissimilarity metric (1 - r) and complete linkage. Intra-tissue clustering was used exclusively for visualization and pattern discovery and was not interpreted as evidence of discrete regulatory modules. For global (whole-body) MAT coordination analysis, clusters were identified by applying a fixed dendrogram height threshold, selected to delineate groups of transcripts exhibiting broadly coordinated temporal expression across tissues. Inter-tissue coherence, synchronization strength, and representative matrices were computed in the same manner as intra-tissue analyses but using MATs across all pairwise tissue combinations. Inter-tissue, tissue-level network visualization was conducted using Cytoscape (v3.10.2).^45^

#### Global (whole-body) mitochondrial-associated transcript synchronization analysis

Global (whole-body) MAT synchronization was quantified using a synchronization score computed for each MAT across all 22 tissues. The score was defined as the sum of all inter-tissue pairwise Pearson correlation coefficients (r) reflecting the similarity of its 24 h expression profiles across tissues. Transcripts exhibiting highly concordant temporal patterns across tissues therefore received higher scores, whereas transcripts showing phase divergence or tissue-specific timing were proportionally down-weighted by negative correlations. To establish a biologically grounded reference point for highly coordinated (“clock-like”) temporal behavior, a curated list of core circadian rhythm genes was used to identify the empirical inflection point corresponding to a major decline in synchronization score (y = 60). This threshold was then applied to the MAT dataset to identify globally coordinated MATs under each dietary condition (ALF and TRF). Overlap analysis between ALF and TRF MAT sets was performed by direct comparison of shared gene symbols to assess diet-dependent shifts in whole-body MAT coordination. For visualization of whole-body MAT temporal waves, the correlation and clustering procedures described above were reapplied, but restricted to MATs exceeding the synchronization threshold.

#### Mitochondrial-associated transcript specific pathway analysis

A custom pathway analysis pipeline was developed using the Mouse MitoCarta3.0^33^^,^^49^^,^^50^ database to functionally annotate MAT temporal waves. The MitoCarta3.0 dataset includes all known mitochondrial-associated genes with curated multi-level pathway annotations (“terms”), and individual genes may be associated with multiple terms depending on annotation granularity. Each pathway term was quantified for its baseline frequency across the full MitoCarta3.0 dataset. Genes were reduced to their associated terms at any annotation level, excluding the broad category “Metabolism”, which was omitted due to limited interpretive specificity. For pathway enrichment, each cluster of globally synchronous MATs was analyzed to identify the top 5 terms ranked by i.) absolute term frequency and ii.) relative term ratio (number of term occurrences within the MAT cluster divided by total occurrences across MitoCarta3.0). Term frequency emphasized dominant biological processes, whereas term ratio highlighted more specific or underrepresented mitochondrial pathways enriched within each temporal wave. All analyses were performed in R (version 4.3.2) and the complete code used for this analysis has been deposited at (https://doi.org/10.6084/m9.figshare.30594017) for public use. The repository contains custom scripts, associated data tables, documentation demonstrating their use for pathway analysis, and a record of relevant package versions and dependencies.

#### Transcription factor enrichment analysis

To provide regulatory context for globally synchronous MAT waveforms, transcription factor (TF) enrichment analysis was performed using ChEA3.^34^ For each global MAT wave cluster identified under ALF and TRF conditions, gene symbols were submitted as input to the ChEA3 web interface (https://maayanlab.cloud/chea3/), which integrates multiple TF-target gene reference libraries derived from ChIP-seq, perturbation, and co-expression datasets. TFs were ranked using the ChEA3 Mean Rank metric, which reflects the average rank of TF across all included libraries. For visualization, the top 10 TFs per wave cluster were reported along with the number and proportion of MATs within each cluster associated with a given TF. This analysis was used to describe transcriptional association patterns and does not imply direct or rhythmic TF regulation.

#### Data acquisition for *Coq10b* transcript dynamics across metabolic and genetic perturbations

Liver transcriptomic datasets from male mice were obtained primarily from the CircadiOmics: Circadian Omic Data Web Portal^36^^,^^37^ (https://circadiomics.igb.uci.edu) for analysis of *Coq10b* transcriptional dynamics across diverse metabolic and genetic perturbations. All datasets were used as processed in their respective source studies, without additional min-max normalization, as preservation of expression amplitude was required for these analyses. Dietary manipulations included: isocaloric feeding restricted to either the light or dark phase (GSE266543)^21^; 24 h fasting versus *ad libitum* feeding (GSE107787)^17^; and standard chow vs. high-fat diet feeding (GSE52333).^15^ Genetic circadian perturbations included whole-body *Bmal1*-KO (pan clock-KO, Liver-KO), liver-specific clock reconstitution (Liver-RE), and SCN-restricted clock rescue models (*Bmal1*^*KO,SCN−RE*^), accessed via the CircadiOmics datasets “MOUSE SASSON LIVER KO”, “MOUSE SASSON LIVER RE”, “MOUSE LIVER SCN RE WT”, “MOUSE LIVER SCN RE KO”, and “MOUSE LIVER SCN RE RE”. Additional diet-clock interaction data were obtained from Chaix A. et al. (GSE102072).^12^ A summary of all public datasets used for contextual *Coq10b* analyses, including dietary conditions, genetic models, dietary paradigm, and sampling parameters, is provided in Table S2. Full experimental details for each dataset are provided in the corresponding original publications.

#### *Coq10b* transcriptional regulatory analysis

Publicly available ChIP-seq and epigenomics datasets were examined to characterize the transcriptional regulatory landscape surrounding the *Coq10b* transcription start site (TSS). Data were accessed through the ChIP-Atlas web portal^46^^,^^47^^,^^48^ (https://chip-atlas.org) and the UCSC Genome Browser^41^ (GRCm38/mm10 assembly; http://genome.ucsc.edu). ChIP-Atlas was queried for mouse liver ChIP-seq experiments using the “Target Genes” function and restricted to a ±2 kb window of the *Coq10b* TSS. Binding profiles for general transcriptional markers (RNA polymerase II, H3K27ac, H3K4me1, H3K4me3, CTCF), circadian transcription factors (ARNTL/BMAL1, CLOCK, NR1D1, RORC, DBP, NFIL3, BHLHE40), and metabolic transcription regulators (PPARA, PPARG, KLF15, ChREBP, HNF4a, CREB1, CEBPB) were extracted. Only datasets derived from mouse liver tissue were included. Additional epigenomic features – including CpG islands, bisulfite sequencing methylation profiles, ATAC-seq and DNase-seq accessibility, and ENCODE candidate *cis*-regulatory elements (cCREs) – were visualized using the UCSC Genome Browser. All analyses represent a qualitative integrative assessment of publicly available datasets; no quantitative reprocessing of sequencing data was performed.

### Quantification and statistical analysis

All statistical analyses were performed in R (version 4.3.2) or GraphPad Prism (version 10.6.1). Pairwise relationships between MATs were assessed using Pearson correlation coefficients (r) calculated across 24-h temporal expression profiles. For intra- and inter-tissue analyses, correlation matrices were generated for all MAT pairs, and synchronization strength was quantified as the mean absolute correlation coefficient (|r|) across each matrix. Signed correlation values were retained for analyses focused on phase alignment. Statistical significance of pairwise correlations was determined using standard parametric methods implemented in R, generating *p*-values for each correlation coefficient that were used as descriptive measures of association strength across the full distribution of gene-gene correlations. Because the primary objective of this study was to characterize global coordination patterns rather than to test specific gene-level hypotheses, multiple testing correction was not applied. For comparisons of synchronization strength between feeding conditions (ALF and TRF), differences in matrix-average |r| values were computed for each tissue. These values were used to assess relative changes in coordination across tissues rather than perform formal statistical hypothesis testing. Hierarchical clustering of correlation matrices was performed using a correlation-based dissimilarity metric (1-r) with complete linkage. Clustering was used for visualization and identification of coordinated expression patterns. For global (whole-body) analyses, dendrogram height thresholds were applied to define clusters of synchronized MATs. Global synchronization scores for individual MATs were calculated as the sum of all inter-tissue pairwise correlation coefficients (r), reelecting the degree of concordance in temporal expression patterns across tissues. A threshold for defining highly synchronized (“clock-like”) transcripts was established empirically based on the distribution of scores observed for curated circadian rhythm genes. For pathway enrichment analysis, mitochondrial pathway terms derived from MitoCarta3.0 were quantified using both absolute term frequency and relative term ratio within each MAT cluster. These metrics were used for ranking and descriptive composition of pathway representation and were not subjected to inferential statistical testing. Transcription factor enrichment analysis was performed using ChEA3 platform, with transcription factors ranked according to the Mean Rank metric across integrated references libraries. Reported enrichments represent relative rankings and proportions of associated genes within each MAT cluster. For datasets with biological replicates, expression values were averaged across replicates prior to analysis, and statistical comparisons were performed on these aggregated profiles.

## Supplemental information

Document S1. Figures S1–S5 and Table S2

Table S1. ALF and TRF synchronization scores for MATs across all tissues
