# Supplementary material for: Time-restricted feeding enhances cross-tissue temporal coordination of mitochondrial-associated transcripts
Source: iScience. 2026 May 15;29(6):115957. doi: 10.1016/j.isci.2026.115957 (PMC13206650; doi:10.1016/j.isci.2026.115957)
Supplement: Document S1. Figures S1–S5 and Table S2 [file mmc1.pdf]

iScience, Volume *29*

## Supplemental information

# Time-restricted feeding enhances cross-tissue temporal coordination of mitochondrial-associated transcripts

**Dylan C. Sarver, Yaniv Maddahi, Christopher S. Colwell, and Aldons Jake Lusis**

A

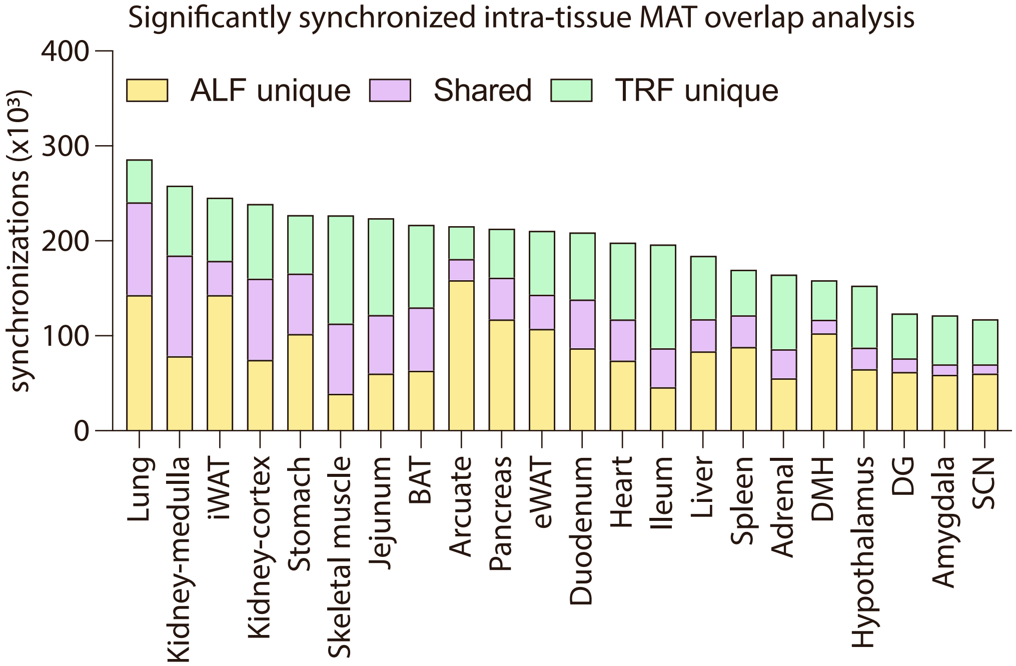


**Figure S1. Overlap analysis of significantly synchronized intra-tissue mitochondrial-associated transcripts across dietary conditions (related to Figure 1).** (A) Number of significantly synchronized intra-tissue MATs per tissue under ALF and TRF. ALF-unique MATs are shown in yellow-orange, TRF-unique in green, and shared MATs in purple.

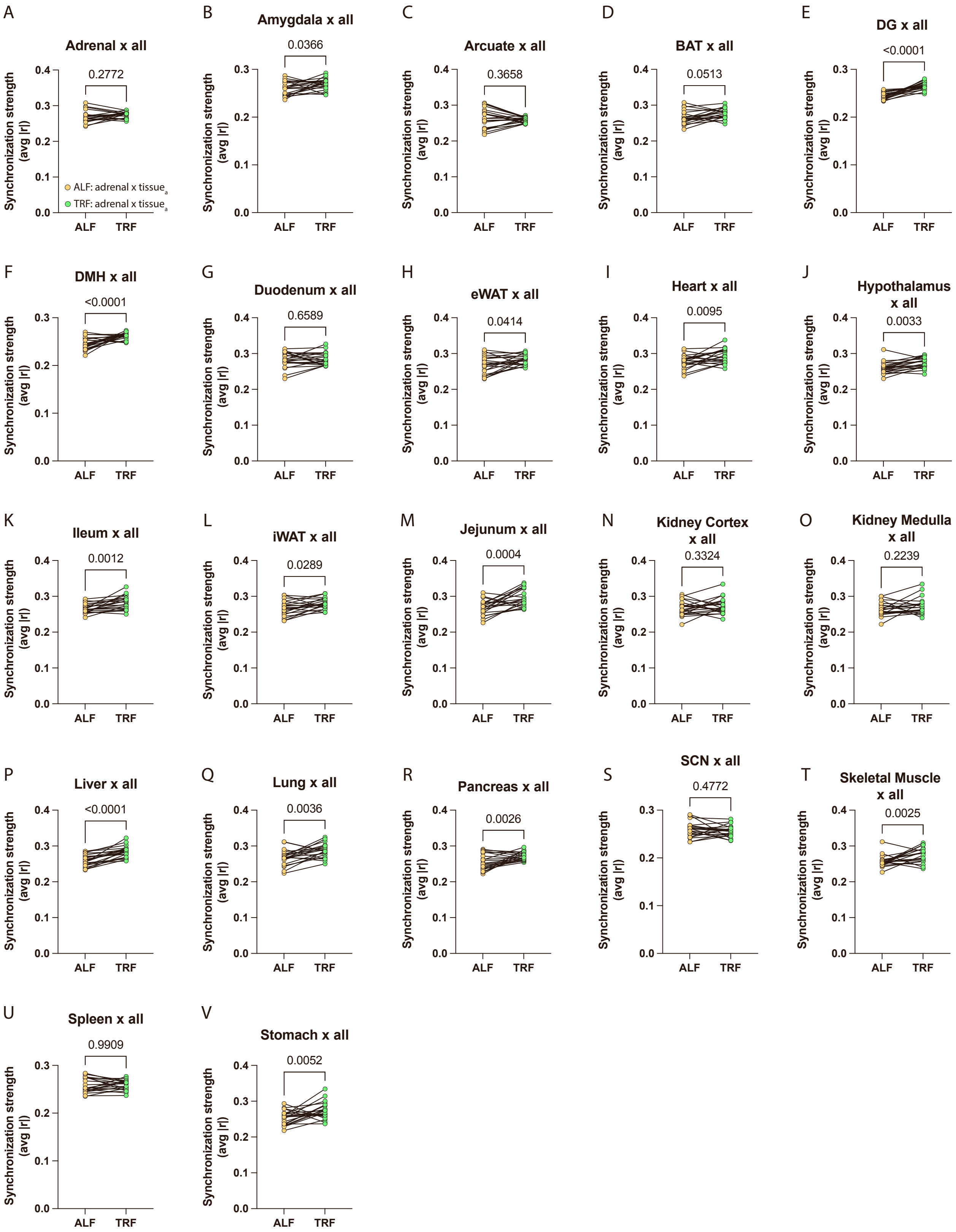

A
Adrenal x all
0.2772
ALF: adrenal x tissue_a
TRF: adrenal x tissue_a
B
Amygdala x all
0.0366
C
Arcuate x all
0.3658
D
BAT x all
0.0513
E
DG x all
<0.0001
F
DMH x all
<0.0001
G
Duodenum x all
0.6589
H
eWAT x all
0.0414
I
Heart x all
0.0095
J
Hypothalamus x all
0.0033
K
Ileum x all
0.0012
L
iWAT x all
0.0289
M
Jejunum x all
0.0004
N
Kidney Cortex x all
0.3324
O
Kidney Medulla x all
0.2239
P
Liver x all
<0.0001
Q
Lung x all
0.0036
R
Pancreas x all
0.0026
S
SCN x all
0.4772
T
Skeletal Muscle x all
0.0025
U
Spleen x all
0.9909
V
Stomach x all
0.0052
Synchronization strength (avg |r|)
0.0
0.1
0.2
0.3
0.4
ALF
TRF

**Figure S2. Inter-tissue analysis of mitochondrial-associated transcript synchronization strength (related to Figure 3).** (A-V) System-wide analysis of MAT synchronization strength across 22 tissues (alphabetical order) under ALF and TRF. Each tissue is connected across dietary states by a black line. Statistical significance was determined using unpaired two-tailed t-tests and is reported numerically.

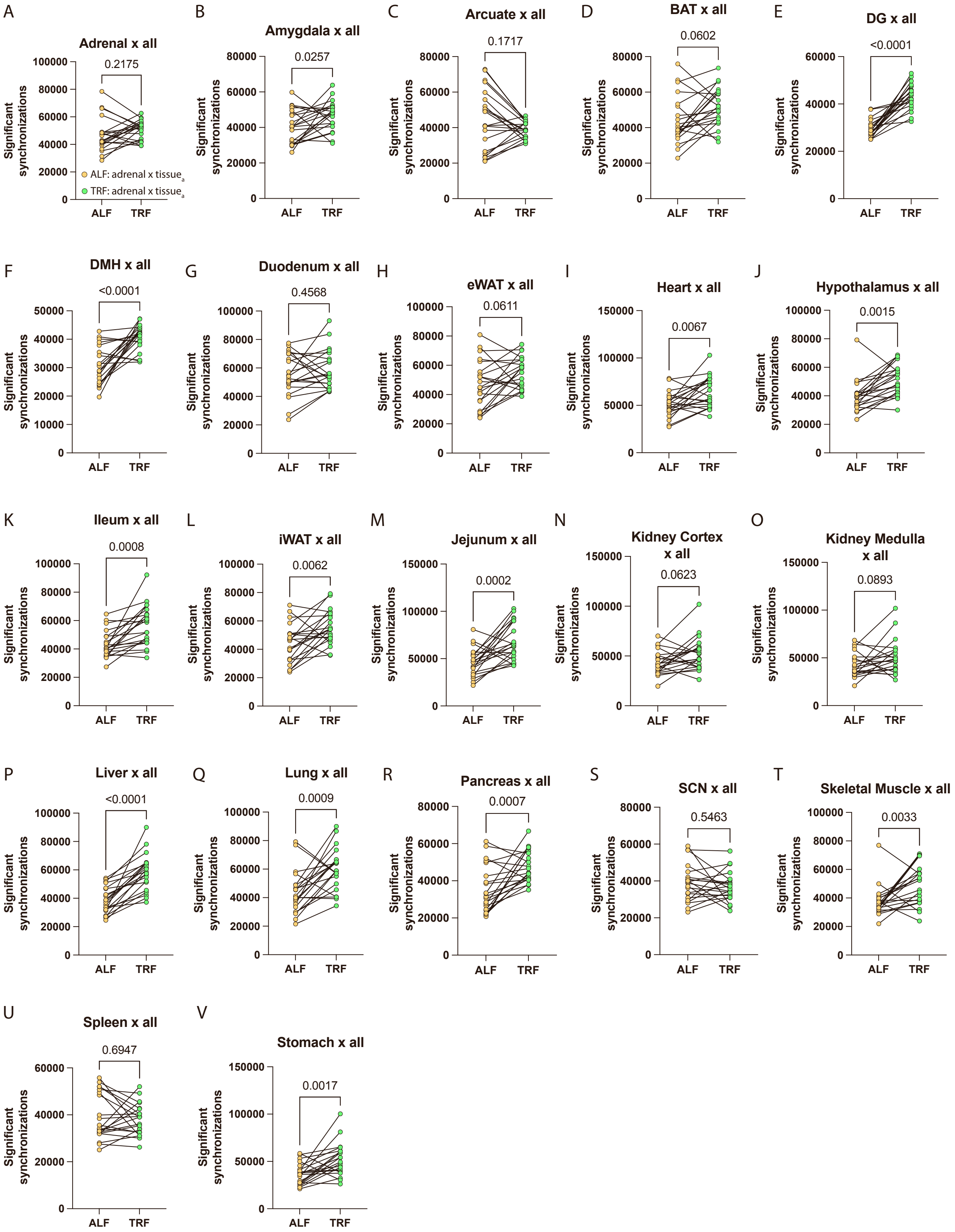
A
Adrenal x all
0.2175
Significant synchronizations
100000
80000
60000
40000
20000
0
ALF: adrenal x tissue$_a$
TRF: adrenal x tissue$_a$
ALF
TRF
B
Amygdala x all
0.0257
C
Arcuate x all
0.1717
D
BAT x all
0.0602
E
DG x all
<0.0001
F
DMH x all
<0.0001
G
Duodenum x all
0.4568
H
eWAT x all
0.0611
I
Heart x all
0.0067
J
Hypothalamus x all
0.0015
K
Ileum x all
0.0008
L
iWAT x all
0.0062
M
Jejunum x all
0.0002
N
Kidney Cortex x all
0.0623
O
Kidney Medulla x all
0.0893
P
Liver x all
<0.0001
Q
Lung x all
0.0009
R
Pancreas x all
0.0007
S
SCN x all
0.5463
T
Skeletal Muscle x all
0.0033
U
Spleen x all
0.6947
V
Stomach x all
0.0017

**Figure S3. Inter-tissue analysis of mitochondrial-associated transcripts with significant synchronizations (related to Figure 3).** (A-V) System-wide analysis of statistically significant MAT synchronizations across 22 tissues (alphabetical order) under ALF and TRF. Each tissue is connected across dietary states by a black line. Statistical significance was determined using unpaired two-tailed t-tests and is reported numerically.

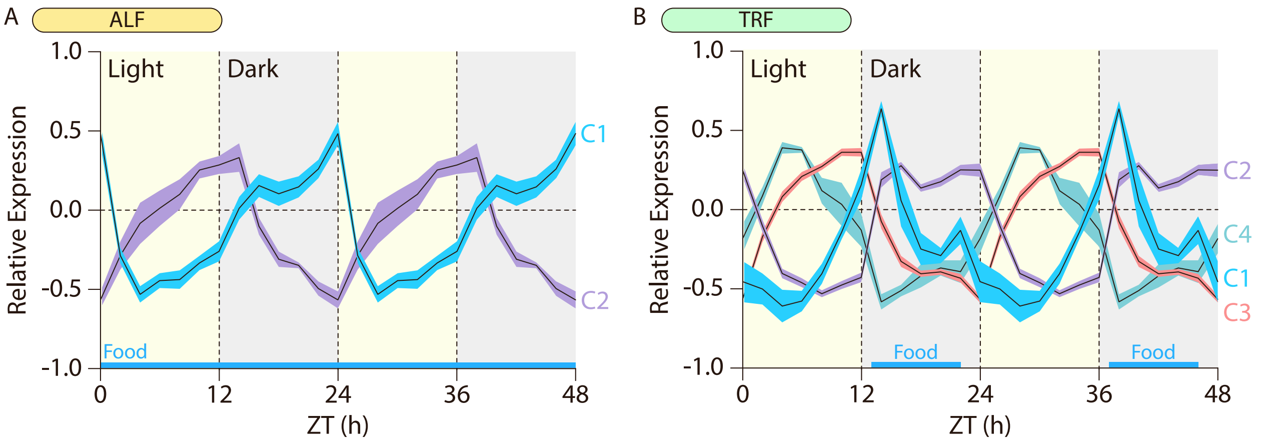


**ALF**

| C1 Gene Symbol (rank) | C2 Gene Symbol (rank) |
|---|---|
| Cry1 (1) | Coq10b (3) |
| Hspd1 (6) | Rsad1 (4) |
| Pdk4 (7) | Mthfd1l (5) |
| Fastkd5 (9) | Slc25a33 (8) |
| Mgme1 (10) | Lias (14) |
| Abca9 (11) | Trub2 (15) |
| Mmaa (12) | Slc25a16 (8) |
| Mcl1 (13) | Sdr39u1 (19) |
| Opa3 (16) | Tamm41 (20) |
| Hspe1 (17) | Hoga1 (21) |
| Xpnpep3 (22) | |
| Ak3 (23) | |

**TRF**

| C1 Gene Symbol (rank) | C2 Gene Symbol (rank) | C3 Gene Symbol (rank) | C4 Gene Symbol (rank) |
|---|---|---|---|
| Coq10b (1) | Cry1 (2) | Tk2 (5) | Adck5 (22) |
| Mthfd1l (20) | Fastkd5 (3) | Slc25a16 (8) | Slc8b1 (43) |
| Alas2 (26) | Mgme1 (4) | Pink1 (11) | E130311K13Rik (45) |
| Mcl1 (27) | Hspd1 (6) | Tamm41 (12) | Pdk4 (54) |
| Slc25a33 (63) | Mmaa (7) | Prkn (14) | Clpx (62) |
| | Opa3 (9) | Fbxl4 (17) | Nlrx1 (76) |
| | Hspe1 (10) | Rsad1 (18) | |
| | Slc25a25 (15) | Cpt1a (21) | |
| | Nudt5 (16) | Nmnat3 (25) | |
| | Noct (19) | Sfxn3 (29) | |
| | Mtrf1l (23) | Coq8a (31) | |
| | Alkbh1 (24) | Bnip3 (32) | |
| | Ndufaf4 (28) | Taz (34) | |
| | Ears2 (30) | Pnpo (36) | |
| | Mrpl19 (33) | Plscr3 (38) | |
| | Mrrf (35) | Casp9 (42) | |
| | Atpsckmt (37) | Parl (44) | |
| | Timmdc1 (39) | Polg2 (47) | |
| | Spire1 (40) | Nipsnap2 (48) | |
| | Slc25a29 (41) | Cmc1 (49) | |
| | Pde12 (46) | Hoga1 (51) | |
| | Slc25a38 (50) | Fahd2a (52) | |
| | Rdh13 (55) | Pccb (53) | |
| | Mars2 (56) | Stx17 (57) | |
| | Trmt10c (60) | Mocs1 (58) | |
| | Slc25a19 (61) | Sirt5 (59) | |
| | Mrps25 (65) | Pex11b (64) | |
| | Twnk (66) | Selenoo (68) | |
| | Agpat5 (67) | Slc25a34 (69) | |
| | Coq5 (72) | Trub2 (70) | |
| | Nif3l1 (74) | Cyb5r3 (71) | |
| | Pdf (78) | Tomm20 (73) | |
| | Xpnpep3 (79) | Lias (75) | |
| | Rhot1 (80) | Eci2 (77) | |
| | Sod2 (81) | Glod4 (82) | |
| | Pgs1 (83) | Ehhadh (84) | |
| | Ccdc127 (87) | Lyrm2 (85) | |
| | Yars2 (88) | Cbr4 (86) | |
| | Tmem177 (89) | | |

**Figure S4. Whole-body mitochondrial-associated transcript waves of globally synchronous genes (related to Figure 4).** Whole-body transcriptional waveforms of MATs identified as globally synchronous under ALF (A) and TRF (B). Food timing is indicated along the x-axis, and genes within each waveform are listed with their synchronization score rankings. Data are represented as mean ± SEM.

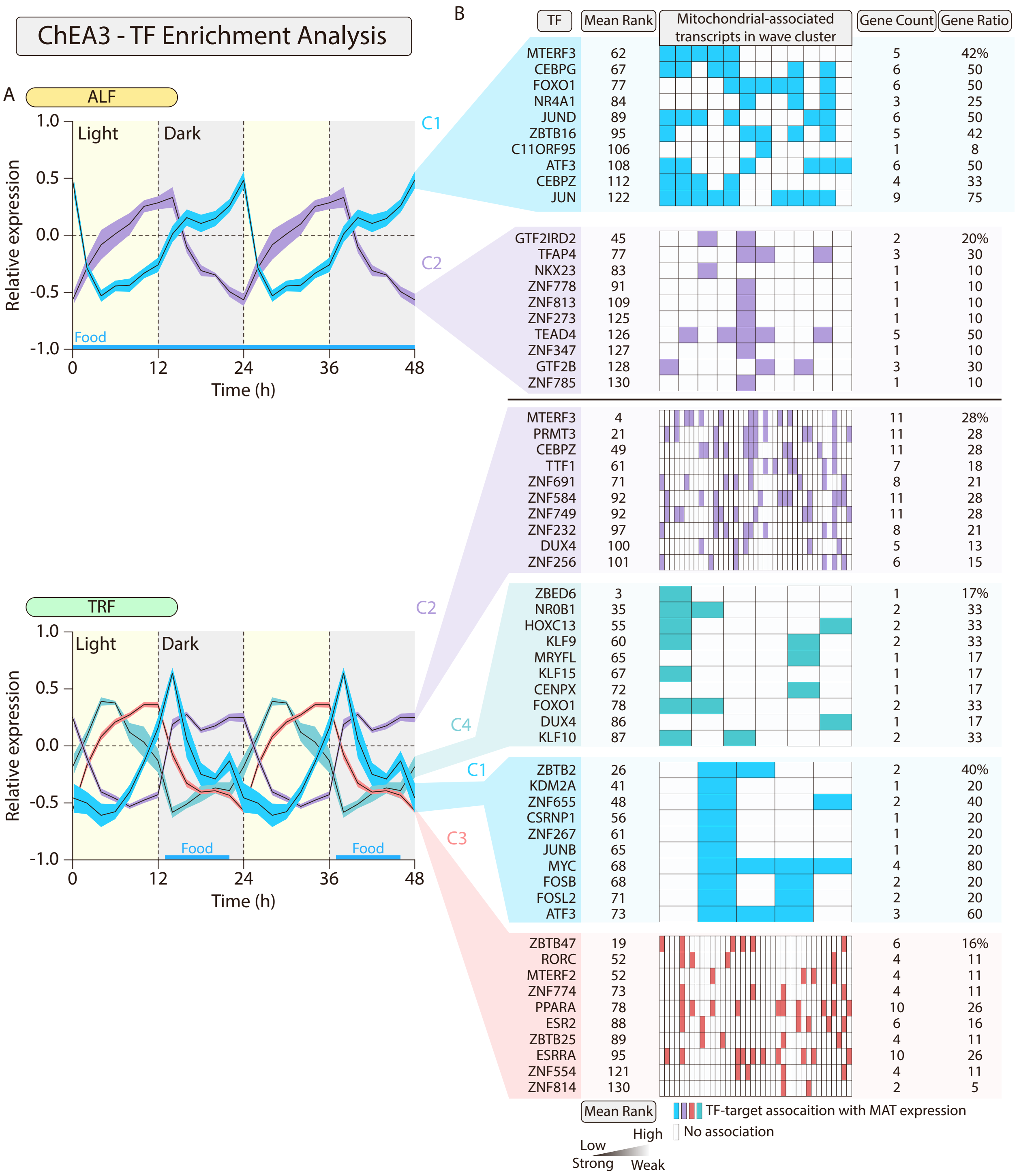


**Figure S5. Transcription factor enrichment analysis of globally synchronous mitochondrial-associated transcript waves under ALF and TRF (related to Figure 4).** (A) Whole-body transcriptional waveforms of MATs identified as globally synchronous under ALF (top) and TRF (bottom). Food timing is indicated along the x-axis, and expression profiles are shown across two consecutive days. Data are represented as mean ± SEM. (B) Transcription factor (TF) enrichment analysis of each globally synchronous MAT waveform cluster performed using ChEA3. Shown from left to right are the top 10 TFs associated with each waveform, the ChEA3 Mean Rank for each TF (lower values indicate stronger and

more consistent enrichment across integrated datasets), a binary TF-MAT association matrix, Gene Count, and Gene Ratio. The TF-MAT association matrix indicates whether a given MAT within the waveform cluster is associated with the indicated TF according to ChEA3 (colored boxes); white boxes indicate no inferred association. Gene symbols are omitted from the x-axis for clarity. Gene Count denotes the number of MATs within the waveform cluster associated with a given TF, and Gene Ratio indicates the proportion of associated MATs relative to the total number of genes in the waveform. These analyses identify partial and overlapping TF associations with globally synchronous MAT waves and do not imply direct or rhythmic regulatory activity.

| Dataset/GEO Accession | Comparison | Reference | PMID | Diet Type | Feeding Paradigm | Sample Size, Resolution, and Duration | Sex | Strain | Age | Tissues Analyzed |
|---|---|---|---|---|---|---|---|---|---|---|
| GSE266543 | Isocaloric TRF-6h Light vs TRF-6h-Dark | Acosta-Rodriguez V., et al. 2024 | 39046875 | Standard chow | TRF-Light vs TRF-Dark | n=2/time point, 4h, 48h | Male | C57BL/6J | 6 months | Liver |
| GSE107787 | ALF vs 24h Fasted | Kinouchi K., et al. 2018 | 30566858 | Standard chow | ALF vs Fasted | n=4/time point, 4h, 24h | Male | C57BL/6J | 2 months | Liver |
| GSE52333 | Standard chow vs HFD | Eckel-Mahan K., et al. 2013 | 24360271 | Standard chow and HFD | ALF-Standard chow vs ALF-HFD | n=3/time point, 4h, 24h | Male | C57BL/6J | 4 months | Liver |
| CircadiOmics "MOUSE SASSON ILLUMINA RE" | Pan-clock-KO, Liver-clock restoration | CircadiOmics | N/A | Standard chow | ALF | n=3/time point, 4h, 24h | Male | C57BL/6J | unknown | Liver |
| CircadiOmics "Mouse Liver SCN RE" | Pan-clock-KO, SCN-clock restoration | CircadiOmics | N/A | Standard chow | ALF | n=3/time point, 4h, 24h | Male | C57BL/6J | unknown | Liver |
| GSE102072 | ALF vs TRF-9h-Dark | Chaix A., et al. 2019 | 30174302 | HFD | ALF vs TRF-9h-Dark | n=2/time point, 4h, 24 | Male | C57BL/6J | 5 months | Liver |
| GSE102072 | ALF-Bmal1-LKO vs TRF-9h-Dark-Bmal1-LKO | Chaix A., et al. 2019 | 30174302 | HFD | ALF vs TRF-9h-Dark | n=2/time point, 4h, 24 | Male | C57BL/6J | 5 months | Liver |
| GSE102072 | ALF-Nr1d1/1-LDKO vs TRF-9h-Dark-Nr1d1/2-LDKO | Chaix A., et al. 2019 | 30174302 | HFD | ALF vs TRF-9h-Dark | n=2/time point, 4h, 24 | Male | C57BL/6J | 5 months | Liver |
| GSE102072 | ALF-Cry1/2-WBKO vs TRF-9h-Dark-Cry1/2-WBKO | Chaix A., et al. 2019 | 30174302 | HFD | ALF vs TRF-9h-Dark | n=2/time point, 4h, 24 | Male | C57BL/6J | 5 months | Liver |

**Table S2. Transcriptomic datasets used for contextual analysis of *Coq10b* regulation across dietary and circadian perturbations (related to Figure 6 and STAR Methods).** Summary of all publicly available mouse liver transcriptomic RNA-sequencing datasets used to contextualize *Coq10b* temporal expression dynamics in Figure 6. For each dataset, the associated publication, dietary composition, feeding paradigm, genetic background, sex, strain, sampling design, and tissues analyzed are listed. All datasets were obtained from the CircadiOmics portal or original source studies and were analyzed as processed in their respective publications, without additional normalization, to preserve relative expression levels and circadian amplitude. Detailed experimental procedures, housing conditions, and associated physiologic data are provided in the corresponding original studies.
